# Perioperative Management Strategies for Fracture Surgery in Patients With Traumatic Pneumothorax: A Retrospective Case Series

**DOI:** 10.7759/cureus.109760

**Published:** 2026-05-27

**Authors:** Kazuyasu Aoki, Taku Mayahara, Tomohiro Katayama, Kazuyoshi Matsuura, Hiroyuki Arai, Eisaku Maruo, Yuki Higashi

**Affiliations:** 1 Department of Emergency and General Medicine, Kobe Ekisaikai Hospital, Kobe, JPN

**Keywords:** chest tube, fracture surgery, perioperative management, positive pressure ventilation, traumatic pneumothorax

## Abstract

Introduction: When patients with traumatic pneumothorax require fracture surgery, clinicians must decide how to manage the pneumothorax perioperatively. Options include chest tube placement for the pneumothorax itself, prophylactic drainage before surgery, proceeding with general anesthesia and positive pressure ventilation (PPV) without drainage, using alternative anesthetic techniques to avoid PPV, or delaying surgery until the pneumothorax improves. Despite the clinical relevance of this scenario, few studies have described how these strategies are selected in actual perioperative practice.

Methods: We retrospectively reviewed patients with traumatic pneumothorax who underwent fracture surgery at a community hospital in Japan between January 2013 and December 2024. Patient characteristics, pneumothorax size, associated injuries, chest tube management, anesthetic technique, timing of surgery, and perioperative outcomes were extracted from medical records.

Results: Nine patients were identified. Management strategies varied and included early chest tube placement for clinical indications independent of surgery (two cases), prophylactic preoperative chest tube placement (one case), regional anesthesia without PPV (one case), and general anesthesia with PPV without chest tube placement (five cases). Among the five patients who underwent general anesthesia with PPV without chest tube placement, those with minimal pneumothoraces underwent early surgery, whereas those with larger pneumothoraces had surgery delayed until improvement was confirmed. No intraoperative tension pneumothorax occurred. One patient with multiple displaced rib fractures projecting into the thoracic cavity developed postoperative pneumothorax progression requiring delayed chest tube drainage.

Conclusions: Perioperative management of traumatic pneumothorax in patients undergoing fracture surgery was highly individualized. Pneumothorax size, rib fracture morphology, pneumothorax trajectory, fracture site, surgical urgency, and the availability of regional anesthetic techniques that avoid PPV should be considered in multidisciplinary decision-making. Further studies are needed to define the risk of clinically significant pneumothorax progression during intraoperative PPV.

## Introduction

Perioperative management of fracture surgery in patients with traumatic pneumothorax poses a clinical challenge, particularly when the pneumothorax is small enough that chest tube drainage is not immediately indicated. In these cases, if general anesthesia with positive pressure ventilation (PPV) is planned, the risk of pneumothorax exacerbation or progression to tension pneumothorax must be considered [1,2]. Several management options exist: prophylactic chest tube placement before surgery, proceeding without drainage with a plan for emergent intraoperative tube thoracostomy if needed, employing anesthetic techniques that avoid PPV, such as regional anesthesia or spontaneous ventilation under general anesthesia, or delaying surgery until the pneumothorax has resolved. Each option carries its own trade-offs. Prophylactic drainage reduces the risk of intraoperative tension pneumothorax but may introduce procedural complications. Emergent intraoperative drainage may be difficult depending on the surgical site and patient positioning, which can restrict thoracic access. PPV-avoidance strategies depend on the patient's respiratory reserve and the suitability of regional techniques for the planned procedure and carry the risk of unplanned conversion to PPV. Delaying surgery allows time for pneumothorax resolution but may be detrimental for fractures requiring early fixation, such as proximal femoral fractures [3].

Despite the clinical relevance of these considerations, few studies have described how often prophylactic chest tube placement is actually selected or how alternative strategies are chosen in real-world perioperative practice. Therefore, the present case series aimed to describe the perioperative management strategies actually employed for patients with traumatic pneumothorax undergoing fracture surgery at our institution and to discuss clinical factors that may inform individualized decision-making.

## Materials and methods

Study design and setting

This was a single-center retrospective case series conducted at an approximately 300-bed community hospital with secondary emergency care capabilities in Japan.

Patient selection

We searched the electronic medical records for patients with documentation of both pneumothorax and fracture between January 2013 and December 2024, cross-referenced these with records of patients who underwent orthopedic surgery at our institution, and manually reviewed all identified records to confirm eligibility. Patients were included if they had a traumatic pneumothorax diagnosed on CT and subsequently underwent fracture surgery during the same hospitalization. Patients in whom the pneumothorax had substantially improved or nearly resolved on imaging prior to surgery were also included, as the perioperative decision-making process in these cases was considered relevant to the study objectives. Eleven patients met these criteria. Two cases that had been previously published as individual case reports [4,5] were excluded from the present analysis to avoid redundant publication. These cases are cited only for contextual comparison in the Discussion section. The remaining nine patients constituted the present case series.

Data collection

Patient demographics and the following variables were extracted from medical records: fracture site, pneumothorax size, presence of subcutaneous emphysema, rib fractures, and hemothorax; chest tube management (presence, timing of insertion relative to injury and surgery, and timing of removal); anesthetic technique; interval from injury to surgery; and perioperative clinical outcomes.

Pneumothorax size measurement

Pneumothorax size was assessed on axial CT imaging and defined as the maximum perpendicular distance between the lung surface and either the chest wall or mediastinum at the largest visible air pocket.

Ethics

This study was approved by the Institutional Review Board of Kobe Ekisaikai Hospital (approval number: 2025-11) and conducted in accordance with the Declaration of Helsinki. The requirement for individual informed consent was waived by the institutional review board because of the retrospective observational design, and an opt-out approach was used. Written informed consent for publication of de-identified clinical information and imaging findings was obtained from the patient’s family member for the case presented in the figures.

## Results

Between January 2013 and December 2024, nine patients with traumatic pneumothorax underwent fracture surgery at our institution. The patient demographics and clinical details are summarized in Table 1. Seven patients were male, and two were female, with a median age of 67 years (range, 42-94). All patients had concomitant rib fractures. The most common surgical procedure was clavicle open reduction and internal fixation (ORIF), performed in six cases; the remaining three cases involved femur ORIF, bipolar hip arthroplasty, and radius ORIF, respectively. Pneumothorax size ranged from <1 mm to 45 mm, with a median of 9 mm. The median interval from injury to surgery was seven days (range, 1-15). Perioperative chest tube management and anesthetic strategies varied considerably among the nine cases (Table 1).

**Table 1:** Patient demographics and perioperative management PTX, pneumothorax; SubQ, subcutaneous emphysema; Hemo, hemothorax; ORIF, open reduction and internal fixation; GA, general anesthesia; PPV, positive pressure ventilation; BPB, brachial plexus block; POD, postoperative day; TBI, traumatic brain injury; CT, computed tomography

Case	Age/sex	Fracture site	Surgery	Initial PTX size (mm)	SubQ	Hemo	Injury to op (d)	Chest tube	Anesthesia	Outcome/notes
Early chest tube										
1	68/M	Clavicle, scapula, ribs	Clavicle ORIF	30	+	+	3	Day 0; removed Day 3	GA + PPV	Tube removed immediately after surgery
2	42/M	Clavicle, ribs	Clavicle ORIF	45	+	+	7	Day 0; removed Day 9	GA + PPV	Uneventful
Prophylactic chest tube										
3	67/M	Clavicle, ribs	Clavicle ORIF	18	-	+	3	Prophylactic Day 3; removed Day 12	GA + PPV	Only case with prophylactic pre-op tube
Regional anesthesia without PPV										
4	81/M	Radius, ribs	Radius ORIF	9	+	-	15	None	BPB only	No general anesthesia required
GA + PPV without chest tube										
5	60/M	Clavicle, ribs	Clavicle ORIF	<1	+	+	1	None	GA + PPV	Uneventful
6	94/F	Clavicle, ribs, femur	Femur ORIF	<1	+	-	1	None; inserted POD 1	GA + PPV	Post-op X-ray: PTX worsening; observed; further progression; tube POD 1; pneumonia developed; died on POD 7
7	79/M	Ribs, femur	Bipolar hip arthroplasty	15	-	+	7	None	GA + PPV	CT Day 5: PTX resolved to 3 mm; surgery proceeded
8	56/F	Clavicle, ribs	Clavicle ORIF	7	+	+	12	None	GA + PPV	Uneventful
9	47/M	Clavicle, ribs, T-spine, skull, TBI	Clavicle ORIF	7	-	+	15	None	GA + PPV	Uneventful

In Cases 1 and 2, chest tubes were placed on the day of injury for clinical indications independent of surgical planning; both patients subsequently underwent surgery under general anesthesia with PPV. In Case 3, a prophylactic chest tube was placed immediately before surgery; this was the only case in which prophylactic drainage was performed specifically to allow safe intraoperative PPV. In Case 4, surgery was performed under brachial plexus block alone, without PPV or chest tube placement. In Cases 5-9, surgery was performed without a chest tube under general anesthesia with PPV. Among these five cases, two patients (Cases 5 and 6) with minimal pneumothoraces (<1 mm) underwent surgery on the day after injury, whereas the remaining three (Cases 7, 8, and 9), whose pneumothoraces measured 7-15 mm, had surgery delayed for seven to 15 days until pneumothorax improvement was confirmed.

**Figure 1:**
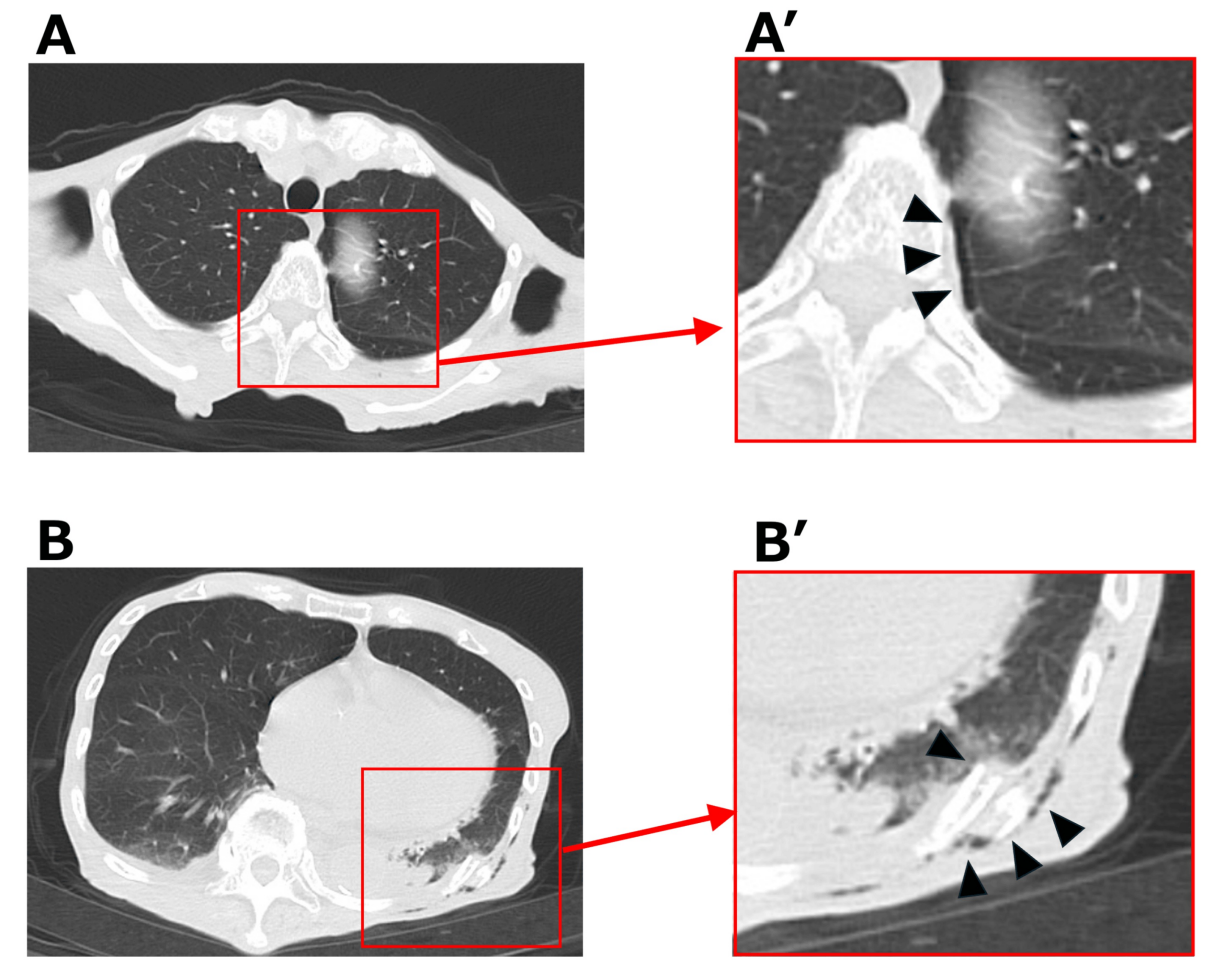
Axial CT on admission (Case 6) (A, A’) A minimal pneumothorax along the mediastinal surface is seen (black arrowheads). The pneumothorax measured <1 mm and was considered unsuitable for prophylactic chest tube placement because there was insufficient pleural air space for safe tube placement. (B, B’) At a different level, subcutaneous emphysema and displaced rib fractures with fragments projecting into the thoracic cavity are demonstrated (black arrowheads). CT, computed tomography

No case of intraoperative tension pneumothorax occurred. In Case 6, a 94-year-old woman with a clavicle fracture and femur fracture required supplemental oxygen (2 L/min via nasal cannula) from admission. Preoperative CT revealed multiple displaced rib fractures with fragments projecting into the thoracic cavity, subcutaneous emphysema, and a minimal pneumothorax measuring <1 mm, which was considered unsuitable for prophylactic chest tube placement because there was insufficient pleural air space for safe tube placement (Figure 1).

**Figure 2:**
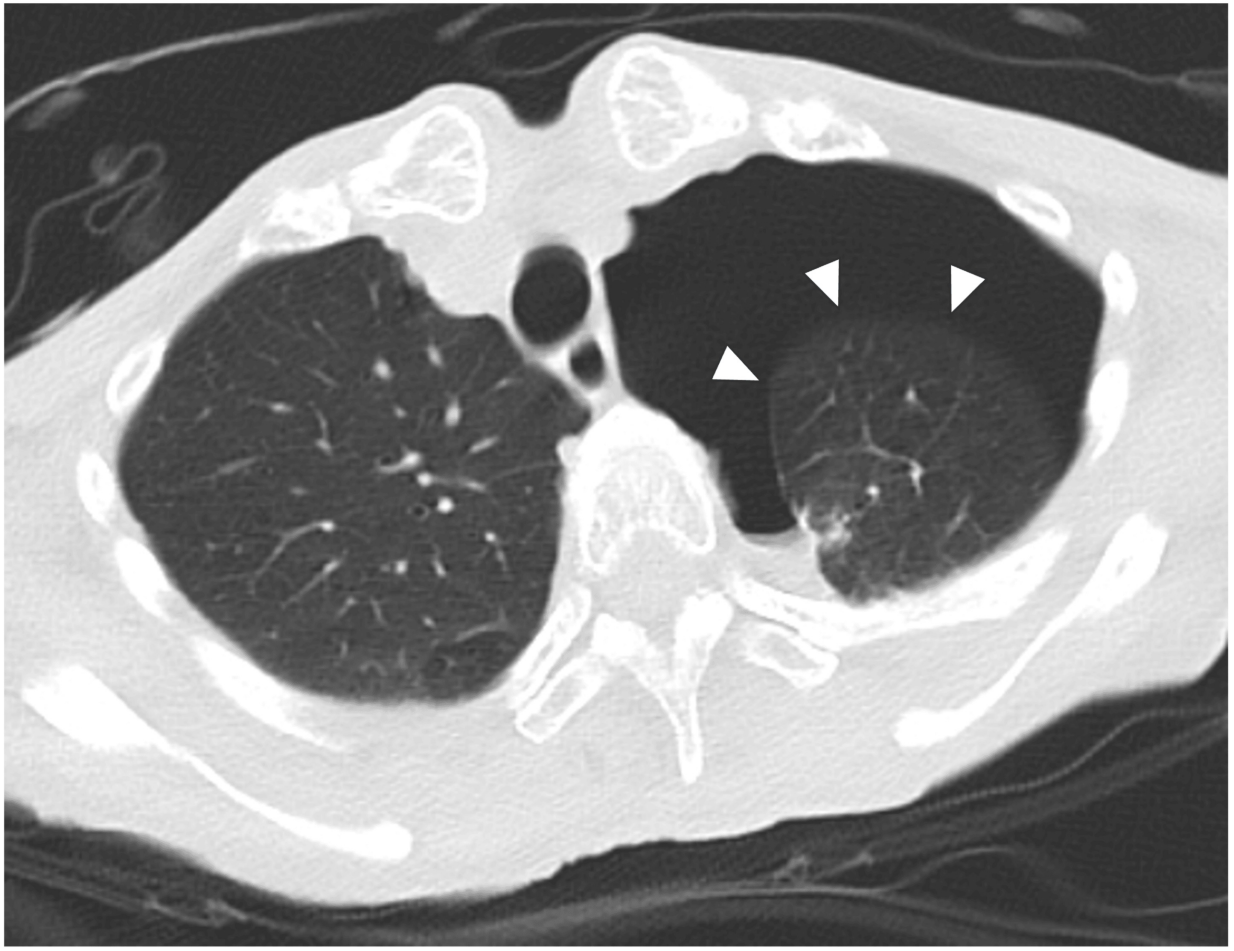
Axial CT on postoperative day one (Case 6) Marked progression of the left-sided pneumothorax is demonstrated (white arrowheads). Chest tube placement was performed following this examination. CT, computed tomography

General anesthesia with PPV was selected in part due to the pre-existing oxygen requirement. The patient underwent femur ORIF (operative time, 102 minutes). A chest radiograph obtained in the operating room prior to extubation revealed worsening of the pneumothorax. Because the patient remained hemodynamically stable without findings suggestive of tension pneumothorax, conservative management with close postoperative monitoring was initially continued. On postoperative day one, oxygen saturation deteriorated, and a repeat CT confirmed further pneumothorax progression (Figure 2).

Chest tube placement was therefore performed. The air leak ceased within two days, and oxygen requirements initially improved. Although the pneumothorax was controlled after chest tube drainage, the patient subsequently developed pneumonia and died on postoperative day seven after a decision was made not to pursue intubation. In all other cases, the perioperative course was uneventful with respect to the pneumothorax.

## Discussion

While trauma guidelines have traditionally recommended tube thoracostomy for traumatic pneumothorax in the setting of PPV [1,2], conservative management has been increasingly studied over the past two decades. In a large single-center observational study of 602 traumatic pneumothoraces, over 90% of conservatively managed patients did not require subsequent tube drainage. Among the 62 patients receiving PPV on admission, the success rate was similarly 90%, with no difference in failure rates between ventilated and nonventilated patients [6]. The OPTICC trial, a multicenter randomized trial of 142 mechanically ventilated patients with occult pneumothoraces, found no significant difference in respiratory distress between drainage and observation groups, although approximately one quarter of observed patients ultimately required drainage, and nearly 40% of those ventilated for more than five days required pleural drainage [7]. The rate of urgent pleural drainage in the observation group was only 6%, suggesting that while delayed nonurgent drainage for progressive effusion or enlargement is not uncommon, acute deterioration necessitating emergency intervention is rare even during prolonged mechanical ventilation. More recently, the Western Trauma Association has proposed a critical decision algorithm recommending observation for pneumothoraces measuring ≤35 mm on CT in hemodynamically stable patients, regardless of ventilation status [8], and the 35 mm rule has been validated as an effective tool for reducing unnecessary tube thoracostomy [9].

However, existing evidence largely derives from emergency, ward, or intensive care settings, and its direct applicability to short-duration intraoperative PPV remains uncertain. The risk of pneumothorax progression during intraoperative PPV, which is typically limited to a few hours, has not been well characterized and is not specifically addressed in current guidelines. Given that prolonged PPV appears to be a risk factor for failure of conservative management, it is plausible that the risk during a time-limited surgical procedure may be lower than that reported in critical care settings. Furthermore, if urgent drainage was required in only 6% of patients observed over days of mechanical ventilation in the OPTICC trial, the likelihood of acute pneumothorax progression necessitating emergency intervention during a surgical procedure lasting only a few hours may be even lower. Nevertheless, this remains to be demonstrated in dedicated perioperative studies. The most directly relevant perioperative report we identified is a conference abstract by Inoue et al., who retrospectively reviewed 50 patients with occult pneumothorax who underwent emergency surgery under general anesthesia at a Japanese trauma center [10]. Among 24 patients who did not receive prophylactic chest drainage, none developed pneumothorax progression, although the non-drainage group had significantly smaller pneumothoraces (median 5.6 mm vs. 11.0 mm, p<0.01), reflecting selection bias inherent in retrospective studies.

In our series, prophylactic chest tube placement before surgery was performed in only one of seven patients who did not require initial drainage. This may reflect a pragmatic risk-benefit assessment by clinicians. Tube thoracostomy carries a reported complication rate of approximately 20%, including insertional injuries, infection, pain, and prolonged hospitalization [11]. These complications may ultimately prove unnecessary if the pneumothorax does not progress during short-duration PPV. However, foregoing prophylactic drainage is not without risk, particularly in clavicle surgery, which accounted for six of nine procedures in this series. Clavicle ORIF is typically performed in the beach-chair position with the ipsilateral arm adducted, which precludes access to the lateral chest wall. If intraoperative tension pneumothorax were to develop, emergent chest tube insertion would need to be performed through the anterior chest within or adjacent to the sterile surgical field, potentially compromising surgical sterility. On the other hand, Case 6 suggests that even a minimal pneumothorax may progress in the perioperative period when accompanied by multiple displaced rib fractures with intrathoracic projection and subcutaneous emphysema. In this case, the pneumothorax was considered unsuitable for prophylactic chest tube placement because there was insufficient pleural air space for safe tube placement; nevertheless, it progressed postoperatively and ultimately required delayed drainage. Although PPV may have contributed to pneumothorax progression, a causal relationship cannot be established from this single case. The pneumothorax improved after chest tube drainage, with cessation of the air leak within two days; however, the patient subsequently developed pneumonia and died. Therefore, although death was clinically attributed to pneumonia rather than uncontrolled pneumothorax, the possibility that transient pneumothorax progression adversely affected the patient’s postoperative course cannot be excluded. This case suggests that pneumothorax size alone may be insufficient for risk assessment, and that rib fracture morphology, subcutaneous emphysema, and limited respiratory reserve may be important considerations even when the initial pneumothorax is minimal.

In the present case series, Case 4 was managed with brachial plexus block alone without PPV or chest tube placement, and the perioperative course was uneventful. In addition, two previously published cases from our institution, which were excluded from the present analysis, illustrate other PPV-avoidance strategies and are cited here only for contextual comparison [4,5]. One case underwent clavicle ORIF under general anesthesia with one-lung ventilation to avoid PPV to the affected lung and had no pneumothorax progression [4]. The other case underwent clavicle ORIF under general anesthesia with spontaneous ventilation using a laryngeal mask airway combined with interscalene brachial plexus block; although pneumothorax progression did not occur, the patient required unplanned postoperative intubation and PPV due to respiratory failure related to phrenic nerve palsy [5]. These cases suggest that PPV-avoidance strategies may reduce exposure of the affected lung to PPV but may also introduce other anesthetic risks.

Based on our findings, we propose that the following clinical factors be considered when formulating perioperative management strategies for patients with traumatic pneumothorax undergoing fracture surgery. First, pneumothorax size and trajectory: larger or progressive pneumothoraces may warrant prophylactic drainage, while small or resolving pneumothoraces may be safely observed. Second, rib fracture morphology: as illustrated by Case 6, displaced fractures with sharp fragments projecting into the pleural space may increase the risk of pneumothorax progression during PPV, even when the initial pneumothorax is small. Third, fracture site and surgical positioning: upper extremity surgery, particularly clavicle fixation in the beach-chair position, may limit thoracic access for emergent intraoperative drainage, whereas lower extremity surgery in the supine position permits easier chest access and may also allow regional anesthesia as the sole technique. Fourth, surgical urgency: fractures requiring early fixation, such as proximal femoral fractures, may necessitate acceptance of a higher-risk strategy rather than prolonged observation [3]. A recent case report described safe elective abdominal surgery performed four weeks after resolution of a traumatic pneumothorax, highlighting that the timing of surgery relative to pneumothorax resolution remains an area without established guidelines [12]. Fifth, the feasibility and risks of alternative anesthetic approaches, including one-lung ventilation, regional techniques, and spontaneous ventilation, should be weighed against the patient's respiratory reserve. These factors do not constitute a formal algorithm but rather a framework for multidisciplinary discussion among surgeons and anesthesiologists to guide individualized decision-making.

This study has several limitations. It was a retrospective case series at a single institution with a small sample size, precluding statistical analysis or identification of independent risk factors. The management strategies were not standardized and reflected individual clinician judgment, introducing selection bias. The specific rationale for each management decision was not consistently documented in the medical records, limiting our ability to characterize the decision-making process beyond what could be inferred from clinical data. Our definition of pneumothorax size, the maximum perpendicular distance from the lung surface to the chest wall or mediastinum, is a simplified measure that does not capture the three-dimensional extent of pneumothorax, and our inclusion of mediastinal-side measurements differs from the chest wall-only measurement used in the 35 mm rule.

## Conclusions

This case series demonstrates that perioperative management of traumatic pneumothorax in fracture surgery patients was highly individualized, with clinicians employing a range of strategies including prophylactic drainage, delayed surgery with observation, regional anesthesia without PPV, and proceeding with PPV without drainage. No case of intraoperative tension pneumothorax occurred, and one patient developed postoperative pneumothorax progression requiring delayed chest tube placement. Although conservative management of traumatic pneumothorax during PPV is increasingly supported in monitored settings, the applicability of these data to short-duration intraoperative PPV remains uncertain. Studies examining the rate of clinically significant pneumothorax progression during intraoperative PPV in patients with known traumatic pneumothorax are needed and would substantially inform perioperative decision-making.
